# Characterization of the complete chloroplast genome sequence of *Sphagneticola calendulacea* (Asteraceae)

**DOI:** 10.1080/23802359.2018.1511846

**Published:** 2018-10-27

**Authors:** Wei Lun Ng, Yubing Zhou, Renchao Zhou, Wei Wu

**Affiliations:** State Key Laboratory of Biocontrol and Guangdong Provincial Key Laboratory of Plant Resources, School of Life Sciences, Sun Yat-sen University, Guangzhou, China

**Keywords:** *Sphagneticola*, Asteraceae, threatened species, complete chloroplast genome, automated assembly

## Abstract

*Sphagneticola calendulacea* is a valuable medicinal herb. With the spread of a congeneric invasive species *Sphagneticola trilobata* in South China, the already vulnerable *S. calendulacea* populations are being threatened further by natural hybridization with the invading species. In this study, we assembled and characterized the complete chloroplast genome of *S. calendulacea* as a resource for future studies on this species. The chloroplast genome was 151,748 bp in size, with a large single-copy (LSC) region of 83,270 bp, a small single-copy (SSC) region of 18,348 bp, separated by two inverted repeat (IR) regions of 25,065 bp each. A total of 134 genes were predicted. Phylogenetic analysis showed close relationship between *S. calendulacea* and *Eclipta prostrata* within the Heliantheae tribe.

*Sphagneticola calendulacea* (L.) Pruski (synonym: *Wedelia chinensis*) is an indigenous perennial herb in South China. This species can be found at riverbanks, coastland, and other moist habitats. With various active constituents like flavonoids, diterpenes, triterpenes, saponins, and phytosterols, *S. calendulacea* has been widely used in traditional medicines (Nomani et al. 2013). Before the introduction of the congeneric invasive species *S. trilobata* (Lowe et al. 2000), *S. calendulacea* was once the only *Sphagneticola* species in South China. In recent years, *S. trilobata* has rampantly spread across South China, threatening the survival of the native *S. calendulacea*. The threats from the invader are many-folds; besides competitive exclusion, natural hybridization with *S. trilobata* is also posing a great threat to *S. calendulacea*. Furthermore, the huge amount of pollen from the invasive species greatly reduces the chance of receiving its own pollen. With widespread hybridization, the F1 hybrid generation has recently emerged as a new competitor (Li et al. 2015; Wu et al. 2013). From our field surveys, many *S. calendulacea* populations have disappeared from their recorded locations. In this study, we characterized the complete chloroplast genome sequence of *S. calendulacea* as a resource for future studies on this species.

We sampled the fresh leaves of *S. calendulacea* from a coastland in Nansha, Guangzhou, China. A voucher specimen was also deposited at the Sun Yat-sen University Herbarium (SYS) with accession number STR-2017-SYS2. Total DNA was extracted using a CTAB protocol, before a library with insertion size of 500 bp was constructed and paired-end (125 bp) sequenced on an Illumina Hiseq2500 platform. Using an *atp*B-*rbc*L gene sequence (GenBank accession JQ065019.1) of *S. calendulacea* as seed, the chloroplast genome assembly was performed using the program NOVOPlasty (Dierckxsens et al. 2017) with ∼4 Gb raw reads as input. The chloroplast genome was then annotated using DOGMA (Wyman et al. 2004) and manually corrected.

The complete chloroplast genome sequence of *S. calendulacea* (GenBank accession KY828438) was 151,748 bp in length, with a large single-copy (LSC) region of 83,270 bp, a small single-copy (SSC) region of 18,348 bp, and a pair of inverted repeat (IR) regions of 25,065 bp each. The genome consisted of 134 predicted genes, including 86 protein-coding genes, 40 tRNA genes, and 8 rRNA genes. The overall GC content was 37.48%.

For phylogenetic analysis, the chloroplast genome sequences of *Adenophora stricta* (Campanulaceae) and 11 representative species of the family Asteraceae were downloaded from the NCBI GenBank database. The 86 protein-coding genes of *S. calendulacea* were then BLASTed against the chloroplast genome sequences of the 12 species. The corresponding orthologs were then extracted and concatenated before sequence alignment using MAFFT v7.307 (Katoh and Standley 2013). After determining the nucleotide-substitution model using the program jModelTest 2 (Darriba et al. 2012), a maximum likelihood tree was constructed with the GTR + G model implemented in the program RAxML (Stamatakis 2014), with *A. stricta* as outgroup. In the phylogenetic tree, *S. calendulacea* was found to be sister to *Eclipta prostrata* (KU361242) within the Heliantheae tribe with strong bootstrap support (Figure 1).

**Figure 1.**
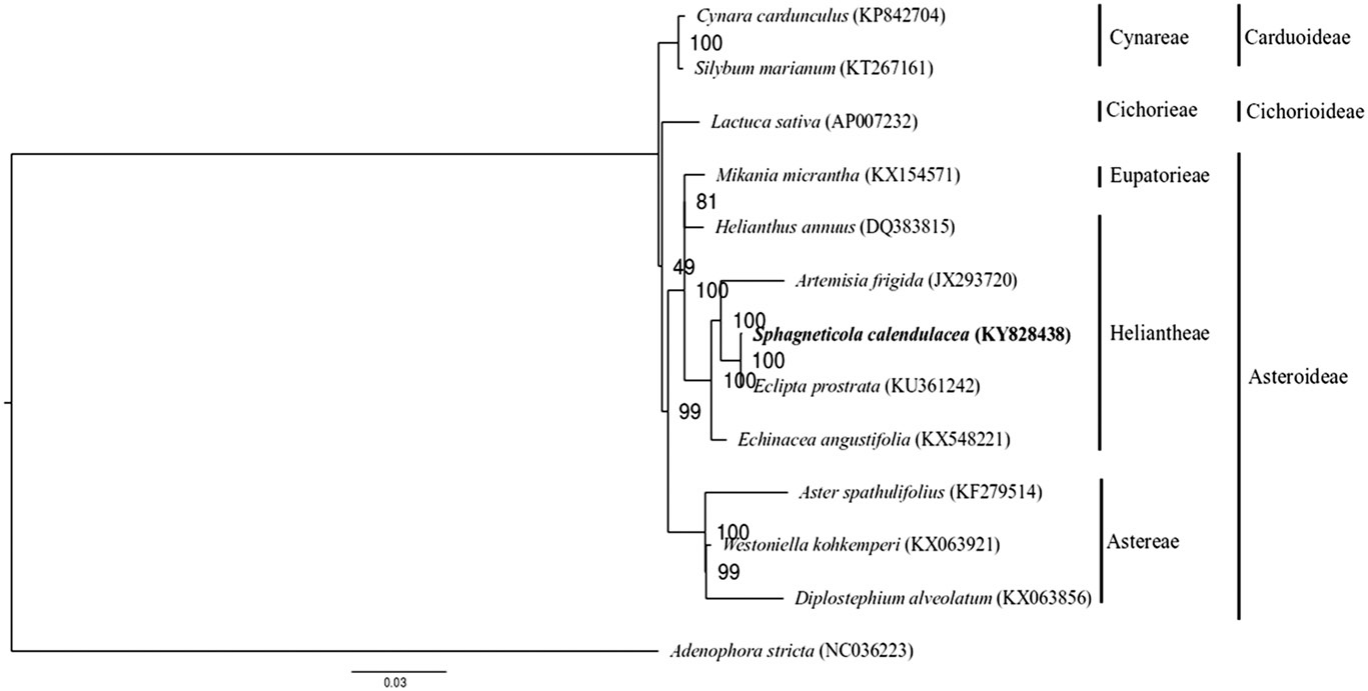
Maximum likelihood tree of *S. calendulacea* and other Asteraceae species based on 86 protein-coding genes, with *A. stricta* as outgroup. Bootstrap support values based on 1000 replicates are shown next to the nodes. Scale in substitutions per site.
